# Stratified analysis reveals chemokine-like factor (CKLF) as a potential prognostic marker in the MSI-immune consensus molecular subtype CMS1 of colorectal cancer

**DOI:** 10.18632/oncotarget.9126

**Published:** 2016-05-02

**Authors:** Philip D. Dunne, Paul G. O'Reilly, Helen G. Coleman, Ronan T. Gray, Daniel B. Longley, Patrick G. Johnston, Manuel Salto-Tellez, Mark Lawler, Darragh G. McArt

**Affiliations:** ^1^ Centre for Cancer Research and Cell Biology, Faculty of Medicine, Health and Life Sciences, Queen's University Belfast, Belfast, UK; ^2^ Centre for Public Health, Faculty of Medicine, Health and Life Sciences, Queen's University Belfast, Belfast, UK

**Keywords:** colorectal cancer, gene expression profiling, molecular stratification, relapse risk, chemokine-like factor

## Abstract

The Colorectal Cancer (CRC) Subtyping Consortium (CRCSC) recently published four consensus molecular subtypes (CMS's) representing the underlying biology in CRC. The Microsatellite Instable (MSI) immune group, CMS1, has a favorable prognosis in early stage disease, but paradoxically has the worst prognosis following relapse, suggesting the presence of factors enabling neoplastic cells to circumvent this immune response. To identify the genes influencing subsequent poor prognosis in CMS1, we analyzed this subtype, centered on risk of relapse.

In a cohort of early stage colon cancer (n=460), we examined, *in silico*, changes in gene expression within the CMS1 subtype and demonstrated for the first time the favorable prognostic value of chemokine-like factor (CKLF) gene expression in the adjuvant disease setting [HR=0.18, CI=0.04-0.89]. In addition, using transcription profiles originating from cell sorted CRC tumors, we delineated the source of CKLF transcription within the colorectal tumor microenvironment to the leukocyte component of these tumors. Further to this, we confirmed that CKLF gene expression is confined to distinct immune subsets in whole blood samples and primary cell lines, highlighting CKLF as a potential immune cell-derived factor promoting tumor immune-surveillance of nascent neoplastic cells, particularly in CMS1 tumors. Building on the recently reported CRCSC data, we provide compelling evidence that leukocyte-infiltrate derived CKLF expression is a candidate biomarker of favorable prognosis, specifically in MSI-immune stage II/III disease.

## INTRODUCTION

Until recently, the classification of CRC has been limited to the generation of prognostic signatures based on gene expression profiles developed by supervised analysis for prognosis. Between 2012 and 2014, a number of studies underpinned a comprehensive molecular characterization of CRC [3–7], culminating in the landmark publication of consensus molecular subtypes by the Colorectal Cancer Subtyping Consortium (CRCSC) [8]. The four Consensus Molecular Subtypes (CMS) identified were: CMS1: microsatellite instability (MSI) immune (frequency 14%); CMS2: canonical (37%); CMS3: metabolic (13%) and CMS4: mesenchymal (23%), providing a more granular discrimination of the underlying CRC disease biology and permitting for the first time an attempt at dissecting the clinical utility of these robust subtypes. The CMS1 subtype is characterized by a high mutation burden, due to the abundance of MSI tumors within this subtype, and a high level of immune infiltration. This subgroup has long been associated with a relatively good prognosis in early stage disease. Paradoxically however, while only a relatively small proportion of patients with MSI tumors subsequently relapse, they have the worst overall prognosis [8–11].

Tumor-associated leukocytes (TALs) have previously been associated with favorable prognosis in CRC, particularly when the density and distribution of CD8+ and CD45RO+ memory T cells are assessed [9, 12]. Recently, these findings were investigated using a pan-cancer meta-analytical framework combining gene expression profiles with survival data to assign prognostic value to gene transcripts [13]. Across a variety of cancers, this approach demonstrated an association between an increased abundance of immune-response genes and improved survival, confirming the generally favorable prognostic value of immune infiltration. While the presence of high immune infiltration is generally associated with improved cancer survival rates, paradoxically the presence of certain subpopulations of T cells can also be associated with poor prognosis [14]. The tissue-specific prognostic value of immune cell infiltration was further highlighted when the association between 22 subsets of TALs and patient survival was examined across 25 cancer types [13]. This pan-cancer approach further revealed the reciprocal, and sometimes counter-intuitive, nature of TAL prognostic associations based on cancer type. But while these published studies suggest that particular immune cell subsets are associated with prognosis in specific cancers; they do not take into account the presence of the emerging stratified subtypes within each cancer type.

To address this question, we employed a stratified approach, specifically in the immune-rich CMS1, to examine the prognostic value of individual gene transcription probesets by supervised risk analysis. This approach highlighted the probesets associated with relapse in the adjuvant stage II/III disease setting. Further survival analysis revealed the prognostic value of our findings specifically in the CMS1, thus validating this stratified analysis approach. Finally, we used gene expression profiles to delineate the source of expression of the identified gene across a range of specific human tissues and primary cells.

## RESULTS

### Stratified approach to generate subgroup specific relapse rates

**Figure 1:**
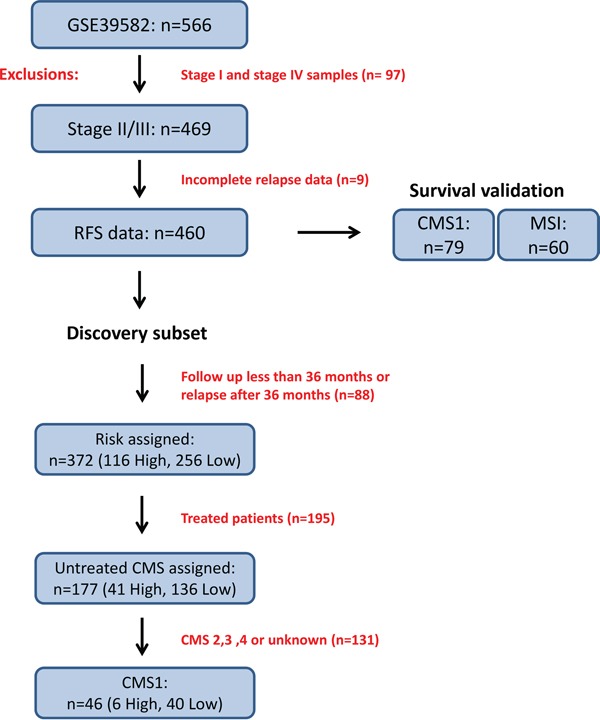
Study overview of discovery and survival validation subsets The data used in this study was obtained from 566 Affymetrix U133 Plus 2.0 patient transcriptional profiles accessed through the NCBI GEO accession number GSE39582. Filtering for stage II/III and complete relapse data reduced this cohort to 460 profiles. The CMS1 specific discovery subset was composed of 46 tumors which fulfilled risk filtering (see Materials and Methods) followed by differential gene expression analysis based on risk classification. The survival validation subset was composed of either the entire cohort, CMS1 specific or MSI specific subgroups of samples. For relapse-free survival analyses, only stage II and III patients were considered, giving 79 transcriptional profiles in the CMS1 or 60 transcriptional profiles in the MSI subgroups.

We analyzed available data from the stage II/III colon cancer tumors in the reference dataset employed by the CRCSC (GSE39582) [7]. Within the overall cohort of n=460 Stage II and III colon cancer cases (Figure 1), 56% were male, 56% were Stage II, 41% were proximal colon tumors, 44% received adjuvant treatment and the mean (SD) age at diagnosis was 68 (13) years (Table 1). Individuals with CMS1 tumors were significantly more likely to have proximal colon tumors (78 v 33%, p<0.001), less likely to receive adjuvant treatment (25 v 48%, p<0.001) and were older (mean 70 v 67 years, p=0.05) compared with individuals with other tumor classifications within the cohort (Table 1). No differences in stage or sex distribution were detected between individuals with CMS1 and other tumor classifications.

**Table 1:** Characteristics of patients in CMS1 (Consensus Molecular Subtype) compared with other tumor subtypes

Characteristic	CMS 1 n=80	All other subtypes^*^ n=380	p-value
**Age, years, mean (SD)**	70.2 (14.6)	67.0 (12.0)	0.05
**Sex, n (%)**			
**Male**	38 (47.5)	220 (57.9)	0.09
**Females**	42 (52.5)	160 (42.1)	
**Tumour stage, n (%)**			
**II**	48 (60.0)	211 (55.5)	0.46
**III**	32 (40.0)	169 (44.5)	
**Tumour location, n (%)**			
**Proximal**	62 (77.5)	125 (32.9)	<0.001
**Distal**	18 (22.5)	255 (67.1)	
**Adjuvant treatment receipt, n (%)** **Yes**	20 (25.0)	182 (47.9)	<0.001

Comparative analysis of age, sex, stage, location and treatment for CMS1 (n=80) patients versus the remaining CMS 2, 3, 4 and unclassified patients (n=380).

* Comprises n=193 CMS2, n=50 CMS3, n=99 CMS4 and n=38 unclassified CMS subtypes.

**Figure 2:**
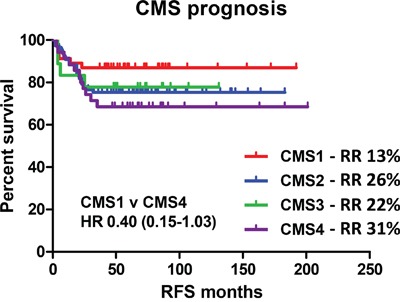
Relapse risk in recently defined consensus molecular subtypes Survival curve using Kaplan-Meier estimation comparing prognosis of CMS1-4 in untreated stage II/III CRC patients (GSE39582). RR indicates the 3 year relapse rate.

We then examined the prognostic value of the four identified subtypes (CMS1-4) based on 3-year relapse risk using a cohort of n=177 untreated stage II/III patient profiles with CMS assignment and complete clinical follow up data (Figure 1). As previously described, there was a trend towards better prognosis in the MSI-immune CMS1 compared to the CMS4 mesenchymal subgroup (3-year relapse rate (RR) of 13% vs. 31%, HR=0.40, 95% CI 0.15 − 1.03) (Figure 2 and Supplementary Table 1).

### Gene expression associated with risk in the CMS1

Using clinical relapse data, we generated an initial discovery subset (detailed in Materials and Methods) resulting in 46 stringently filtered transcriptional profiles specifically assigned to CMS1 (Figure 1). As the frequency of patients assigned to CMS1 in the CRCSC overall cohort was 14%, this collection of 46 patients represents the largest possible publically available CMS1 microarray based dataset from a single cohort. Using an ANOVA model of high risk contrasted with low risk to examine true prognostic biology within CMS1, we generated a list of differentially expressed probesets. This resulted in 55 annotated probesets accounting for 49 unique genes (Table 2). While the majority of these genes were represented only once, Chemokine-Like Factor (CKLF) was represented by all three probesets corresponding to this gene on the microarray platform, highlighting its association with relapse risk.

**Table 2:** Probesets and genes associated with relapse risk in CMS1

Column ID	Gene Symbol	p-value (Risk)	Fold-Change (High Risk vs. Low Risk)
**221058_s_at**	**CKLF**	**0.000302824**	**-1.56349**
242465_at	LOC100505592	0.000332139	−1.78683
**223451_s_at**	**CKLF**	**0.000598022**	**−1.62852**
**219161_s_at**	**CKLF**	**0.0019177**	**−1.56255**
209759_s_at	DCI	0.00192191	−1.51586
213285_at	TMEM30B	0.00219006	−1.86942
229331_at	SPATA18	0.0022411	−2.15593
210002_at	GATA6	0.00239525	−1.95547
203814_s_at	NQO2	0.00317772	−1.88969
232809_s_at	FLT1	0.00388947	−1.58036
221958_s_at	WLS	0.00428383	−1.90076
228360_at	LYPD6B	0.00460096	−2.18477

Column ID	Gene Symbol	p-value (Risk)	Fold-Change (High Risk vs. Low Risk)
205728_at	ODZ1	7.48E-07	1.63545
208148_at	MYH4	1.31E-06	2.36166
214603_at	MAGEA2 /// MAGEA2B	4.30E-05	3.64337
214642_x_at	MAGEA5	9.20E-05	3.02798
235700_at	CT45A1	0.000129371	2.11826
219011_at	PLEKHA4	0.000144152	1.67177
242577_at	LOC389834	0.000198257	1.57463
1553830_s_at	MAGEA2 /// MAGEA2B	0.000294457	2.9941
210467_x_at	MAGEA12	0.00031194	3.36053
220445_s_at	CSAG2 /// CSAG3	0.000344059	2.01273
204086_at	PRAME	0.000353129	2.95165
233092_s_at	LOC100271840	0.00043833	1.78984
214612_x_at	MAGEA6	0.000469934	8.44036
209942_x_at	MAGEA3	0.000595555	8.19105
205563_at	KISS1	0.000821326	1.88576
209733_at	MID2	0.000932513	1.72349
215729_s_at	VGLL1	0.00106856	1.89873
214254_at	MAGEA4	0.00109841	2.16359
244631_at	LOC389834	0.00112161	1.97882
1570445_a_at	LOC643201	0.00116794	1.89823
232195_at	GPR158	0.00120259	2.43469
243683_at	MORF4L2	0.00163486	1.81374
204823_at	NAV3	0.00181836	1.73127
235004_at	RBM24	0.00183565	1.8934
37028_at	PPP1R15A	0.001906	1.72498
210751_s_at	RGN	0.00195509	1.52471
202014_at	PPP1R15A	0.00198168	1.73184
229511_at	SMARCE1	0.00202072	1.70354
210605_s_at	MFGE8	0.00205293	1.7212
224825_at	DNTTIP1	0.00206935	1.64464
227084_at	DTNA	0.00217336	1.69962
236840_at	C12orf56	0.00232896	1.98568
220106_at	NPC1L1	0.00234885	1.71598
1567912_s_at	CT45A1	0.00295615	1.75065
227239_at	FAM126A	0.00346844	2.32878
208075_s_at	CCL7	0.00348212	1.85285
236514_at	ACOT8	0.0037126	1.6944
223138_s_at	DHX36	0.0038878	1.60102
213906_at	MYBL1	0.00395218	2.28576
235561_at	TXNL1	0.00418508	1.77398
229252_at	ATG9B	0.00418806	1.71331
218853_s_at	MOSPD1	0.00482949	1.77617
201023_at	TAF7	0.00499489	1.56954

Differentially expressed probesets and their associated gene name between high and low risk groups in CMS1. Following ANOVA, fold-change +/− 1.5 fold and p-value<0.005 filters were applied resulting in 55 annotated probesets associated with risk in CMS1. Positive fold-change indicates higher expression in high-risk group.

### Clinical relevance of CKLF gene expression in the CMS1

To validate the clinical value of our findings, we examined the stage II/III profiles with the complete data in GSE39582 (Figure 1) [7] by removing all the imposed restraints from the initial discovery subset to create a survival validation subset. Using mean expression of the three probesets representing CKLF, we stratified tumor profiles across all CMSs into high, medium and low, based on CKLF gene expression. When comparing the highest with the lowest levels of CKLF expression, no significant associations with recurrence-free survival were detected in the overall cohort (Table 3 and Supplementary Figure 1A and 1B) with each group overlapping.

**Table 3:** Unadjusted and adjusted analyses of relapse-free survival

	CKLF gene expression^*^		Unadjusted Hazard ratios (95% confidence intervals)	Adjusted^**^ Hazard ratios (95% confidence intervals)
	Number non-events Low/Med/High	Number events Low/Med/High	High v. Low expression	High v. Low expression
**CMS1**				
**Untreated (n=59)**	17/15/19	5/2/1	0.21 (0.04-1.03)	0.19 (0.04-0.96)
**All (n=79)**	20/19/25	7/6/2	0.21 (0.04-1.03)	0.18 (0.04-0.89)
**MSI**				
**Untreated (n=47)**	12/13/16	4/1/1	0.18 (0.02-1.65)	0.19 (0.02-1.85)
**All (n=60)**	15/16/19	5/4/1	0.17 (0.01-1.42)	0.16 (0.02-1.39)
**Entire cohort**				
**Untreated (n=258)**	63/65/68	20/19/23	0.99 (0.54-1.79)	0.93 (0.50-1.73)
**Treated (n=202)**	43/41/41	28/27/22	0.80 (0.45-1.43)	0.90 (0.50-1.62)
**All (n=460)**	106/106/109	48/46/45	0.87 (0.58-1.31)	0.98 (0.64-1.49)

RFS analysis was performed using Cox proportional hazards method in the CMS1, MSI or entire cohort stratified by CKLF expression levels. Analysis was performed both before and following adjustment.

* Cut-offs for low/medium/high CKLF gene expression based on tertile values.

** Adjusted for age, sex, TNM stage, tumour location and adjuvant treatment receipt (in treated analyses only).

**Figure 3:**
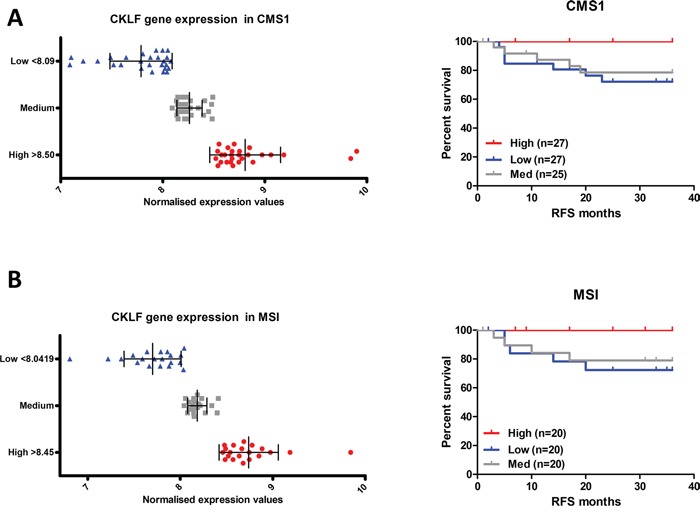
CKLF gene expression is associated with prognosis only in CMS1 **A.** Survival curve using Kaplan-Meier estimation comparing CKLF levels in CMS1 stage II/III CRC patients (GSE39582) **B.** Survival curve using Kaplan-Meier estimation comparing CKLF levels in MSI stage II/III CRC patients (GSE39582).

However, when the CMS1 patient profiles were stratified by CKLF expression separately (CMS1 profiles in the survival validation cohort n=79, Figure 1) a 79% reduced risk of recurrence was evident for the highest compared with the lowest level of CKLF expression (Figure 3A, Table 3 and Supplementary Figure 1C and 1D). This risk of recurrence became statistically significant once confounding factors such as age, stage, tumor location and adjuvant treatment were adjusted for (HR 0.18, 95% CI 0.04-0.89) (Table 3). The CKLF-medium group appeared to have an intermediate survival compared to the other two groups (Figure 3A), indicating that the positive prognostic value of CKLF was restricted to tumors within the highest-expressing tertile. These findings were also demonstrated using Euclidean and Ward clustering with a skewing of high-risk samples towards the low CKLF expression clusters (Supplementary Figure 1E). Using cross tabulation of the patient profiles, we found comparable numbers of BRAF mutants and wildtypes in both the high- (15%) and low-risk (17%) groups, which ruled out the possibility of an inadvertent enrichment for BRAF mutants in the CMS1 high-risk subtype (Supplementary Table 2). In addition, we found no difference in CKLF gene expression across the entire cohort according to CMS (Supplementary Figure 1F), indicating the ubiquitous nature of CKLF gene expression in all CMSs. This trend towards a favorable prognosis in the CMS1 specific CKLF-high group was also observed in a further independent dataset (HR 0.38, 95% CI 0.13-1.13) (GSE14333 n=188, CMS1 n=41), containing a mix of colon and rectal tumor transcriptional profiles, some of which had received adjuvant chemotherapy or neo-adjuvant chemo-radiotherapy treatments regimes (Supplementary Figure 1G).

### Prognostic value of CKLF gene expression in MSI patients

Given that CMS1 is highly enriched for MSI tumors, we performed a further stratification, by selecting only MSI profiles from the stage II/III cohort, regardless of CMS clustering (Figure 1). As expected, these profiles were enriched for the CMS1 samples (49 CMS1 out of 60 MSI tumors − 82%). Patient tumors (n=60) were stratified into three groups based on CKLF gene expression (Figure 3B). Analysis of the high expression compared to the low expression group in the MSI tumor profiles did reflect a similar trend, with an 84% reduced risk, but this result was not significant (HR 0.16, 95% CI 0.02-1.39), indicating that CMS1 transcriptional classification is not simply a marker of MSI status (Table 3, Figure 3B).

### CKLF gene expression is associated with the tumor immune infiltrate

**Figure 4:**
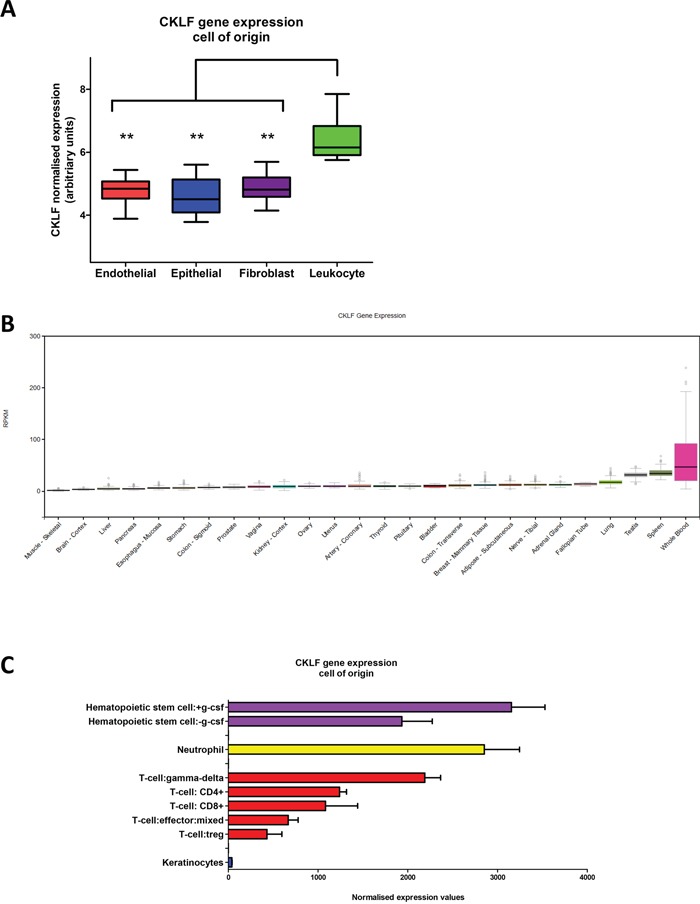
CKLF gene expression within the tumor microenvironment and normal tissue **A.** Box and whisker plot of CKLF expression according to specific endothelial, epithelial, fibroblast or leukocyte cell-of-origin from FACS sorted primary colorectal tumors. **B.** CKLF RNA-seq expression values are shown in RPKM (Reads Per Kilobase of transcript per Million mapped reads), calculated from a gene model with isoforms collapsed to a single gene. No other normalization steps have been applied. Box plots are shown as median and 25th and 75th percentiles; points are displayed as outliers if they are above or below 1.5 times the interquartile range. The data used for the analyses described in this manuscript were obtained from the Genotype-Tissue Expression (GTEx) portal (http://gtexportal.org/home) version V6 in January 2016. **C.** CKLF gene expression from a subset of the Atlas of Human Primary Cells cohort. Bar charts are shown as median CKLF gene expression according to specific lineage and error bars represent standard deviation. ** denotes p<0.005.

Gene set enrichment analysis of the CMS1 group in the CRCSC study revealed an increased expression of genes associated with immune infiltration [8], confirming previous pathology-based associations between MSI and immune infiltrate [10, 11]. Given the distinct structure of the tumor microenvironment (TME) within the CMS1 tumors, we sought to determine if the precise origin of CKLF gene expression was confined to any specific cell type within the TME. To address this question, we utilized a separate microarray dataset obtained from dissociated fresh primary tumors (GSE39396), which had been Fluorescence Activated Cell Sorting (FACS) selected into specific endothelial, epithelial, leukocyte and fibroblast populations [15]. Assessment of the expression profiles according to the cell of origin confirmed that CKLF is significantly associated with tumor infiltrating leukocytes (p<0.005) when compared to the other cell types represented within the TME (Figure 4A).

### CKLF gene expression profile across diverse human tissue and primary cells

While CKLF gene expression is significantly associated with the leukocyte population within CRC tumors, we wished to comprehensively profile its expression levels in other human tissues and cells. Using the recently assembled Genotype-Tissue Expression (GTEx) portal [16, 17] (http://gtexportal.org/home), we examined the gene expression of CKLF (as measured by RNA-seq analysis) across a diverse range of 53 tissues (version V6, January 2016) to investigate the pattern of CKLF gene expression. This analysis revealed high expression of CKLF in whole blood samples, compared to all other tissues represented within the portal (subset shown in Figure 4B). The wide range of expression values observed for CKLF in these whole blood samples, represented by a large spread and outliers, indicates that there is clear variation across patient samples which may be due to the heterogeneous populations represented within whole blood. This finding was confirmed using a further cohort [18] from a panel of 79 human tissues (GSE1133) (subset shown in Supplementary Figure 2).

To further delineate the specific cell type expressing CKLF, we utilized the BioGPS database [19], which captures gene expression profiles across 745 samples representing a diverse range of primary human cells [20] including multiple immune sub-populations detectable in whole blood. Within this comprehensive collection of human cell types, we find CKLF expression levels are highly concordant in all three probesets across the entire cohort of cell types (Supplementary Figure 3). High CKLF gene expression was consistently associated with a small number of immune cell types including hematopoietic stem cells, neutrophils and the anti-tumorigenic γδ T cells (gamma-delta T cells) (Figure 4C).

## DISCUSSION

The recent publication of four consensus molecular subtypes in CRC has provided a framework for clinically relevant stratified-based discovery to be applied in CRC for the first time [8]. Using the reference dataset employed by the CRCSC [7], we specifically selected the MSI-immune CMS1 transcriptional profiles for analysis. Selection of this subtype of tumors in isolation allowed us to identify subtle transcriptional changes associated with relapse risk which would have otherwise been missed across an unstratified patient cohort.

Although stratified medicine does provide a unique opportunity for analyzing specific subtypes, particularly as we move away from the traditional two arm studies to multi-arm, multistage clinical trials, [21] one of the difficulties which will inevitably be encountered with the stratified approach is the analysis of selected small patient cohorts, which are difficult for traditional statistical models to assess. Additionally, as we focus on the good prognostic CMS1 subgroup, the number of relapse events can be very low or even zero in some cases, which is problematic when performing initial proportional hazards fitness analysis. The precise application of stratified medicine will depend on how these statistical issues are resolved, but the model used in this study represents a pipeline approach of how an early discovery phase analysis can yield valuable information and generate hypotheses for validation in focused clinical studies.

This pipeline approach identified CKLF as a good prognostic marker in early stage CMS1 patients, where analysis of relapse rates highlighted the significant prognostic advantage for patients with high levels of CKLF gene expression. These findings indicate the potential for CKLF as a prognostic marker in this stratified group and provide a framework for further hypothesis-driven analysis in ascertaining its true clinical value. Our analysis also ruled out enrichment for BRAF mutations in the high-risk subgroup, as there are similar proportions of BRAF mutations represented in the low-risk group. While a similar prognostic trend for CKLF is also found in MSI tumor profiles, this data fails to reach significance, but may be limited by reduced statistical power in this analysis. It is the limitation of small patient numbers within stratified groups, which may be challenging when examining retrospective unselected cohorts using a stratified approach. This point is further highlighted given the size of the cohort we begin with, containing 566 patient transcriptional profiles, which represents one of the largest publically available single clinical CRC cohort datasets.

The CKLF protein was originally identified from a screen using a monocyte cell line and was found to have a strong chemotactic effect on human neutrophils, lymphocytes and monocytes [22]. CKLF was subsequently found to bind with the chemokine receptor CCR4, while increased CKLF expression was also detected in CD4+ and CD8+ lymphocytes and was associated with T lymphocyte activation [23]. Data presented here confirms these findings, with detection of CKLF gene expression in CD4+ and CD8+ cells, but importantly indicates further increased expression within neutrophils, hematopoietic stem cells (HSCs) and a distinct class of T cells, namely the γδ T cell lineage. The role of neutrophils in CRC development has previously been shown to correlate with an adverse prognosis [24], but these findings have been challenged more recently, as neutrophils can be further subdivided into an anti-tumorigenic (N1) or pro-tumorigenic (N2) phenotype (reviewed in [25]). The CKLF expression level observed in HSCs, which is not maintained in all subpopulations of myeloid and lymphoid progenitor cells, warrants further investigation to determine the role that CKLF plays in this process of immune cell differentiation. Indeed, CKLF is increased following granulocyte colony-stimulating factor (G-CSF) stimulation, which causes mobilization of these cells into the bloodstream [26, 27]. The γδ T cell represents a unique immune population, which has been shown to play a key role in the immune system response to infection, cellular transformation and to tissue damage responses (reviewed in [28]). Although there is a paucity of data on the role of this cell lineage in mediating response to malignancy, [29] γδ T cells do exert a protective role in cancer. A limited number of studies have demonstrated the anti-tumorigenic role of γδ T cells specifically in CRC [30, 31] with increased numbers of γδ T cells being recruited at the earliest stage of tumor initiation [32], while a further study has indicated their ability to protect the host from tumor formation in mouse models of malignancy [33].

While pathology-based studies have highlighted the favorable prognostic value associated with CD8+ T cell infiltration in CRC [9, 12], we now show for the first time that increased expression of CKLF has significant prognostic value in tumors classified as CMS1. It is possible that the increased levels of immune-derived CKLF are a surrogate marker for an increase in a specific type of immune cell infiltrate as suggested by our analyses. The specific anti-tumorigenic roles attributed to these particular classes of immune-infiltrate may also explain the poor prognosis observed in the CKLF-low subgroup of CMS1 tumors. While high levels of CKLF, indicating a high level of specific immune cell types, is sufficient to successfully eliminate these tumors, or at least hold them in a nascent equilibrium state, it is the lack of CKLF expression which suggests that without these immune cell populations within the TME, the neoplastic cells can escape the immune-surveillance and allow disease relapse [34]. These findings would require further detailed molecular pathology analysis in a large cohort of clinically annotated CMS1 tumor samples to validate. A limitation of hypothesis-driven studies such as this one is the requirement of open access to large stratified patient datasets, with matching molecular and clinical data, to allow hypothesis validation. While this issue will continue to limit the application of *in silico* clinical discovery strategies, the responsible but effective sharing of genomic and clinical data as espoused by, for example, the Global Alliance for Genomics and Health [35] to allow complete testing of this approach. Implementing such a molecular pathology “integromics” approach is becoming increasingly relevant in the era of stratified medicine [36–38].

The importance of undertaking a stratification approach for molecular analyses, as described in this study, is further highlighted given the lack of prognostic value attributed to CKLF across the entire unstratified cohort. Without focused supervised analysis of patient transcriptional profiles, research approaches will continue to identify tumor factors which can identify poor prognostic tumor subgroups, such as the mesenchymal CMS4 tumors, based not on relapse risk but on overall tumor subtype. This approach inevitably leads to the assignment of patients into risk categories which are representative of the overall subgroup compared to the general population, but not based on the likelihood of individual relapse *within* the subgroup to which the tumor belongs, a finding which is far more clinically informative. This study also highlights the need for a radical shift in clinical trial design [21] coupled with a harmonized approach to biomarker development and patient stratification [39].

In conclusion, applying the recent consensus molecular subtypes to a large stage II/III patient cohort, we have extended the traditional pathology-based stratification of tumors into immune-high or immune-low tumors which have general prognostic value, with the identification of specific immune-derived factors, enabling us to understand the underlying biology accounting for these prognostic differences. Using this stratified diagnostics approach, we have identified CKLF as a favorable prognostic biomarker of relapse risk in the clinically relevant MSI-immune consensus molecular subtype of CRC. True validation of this type of hypothesis-driven biomarker discovery is reliant on further independent validation of these findings, in a molecular pathology-based analysis of prospective stratified clinical trial material.

## MATERIALS AND METHODS

### Independent datasets

Gene expression profiles from independent CRC datasets were downloaded from NCBI Gene Expression Omnibus (GEO) (http://www.ncbi.nlm.nih.gov/geo/) under accession numbers GSE39582, GSE14333, GSE39396 and GSE1133. GSE39582 contains 566 stage I-IV tumor profiles from a large CRC series, of which 460 stage II/III profiles are utilized in this study. GSE14333 contains 188 Dukes stage B/C profiles from mixed colon and rectal tumors. GSE39396 contains microarray profiles from fresh colorectal specimens where Fluorescence Activated Cell Sorting (FACS) selected cells into specific endothelial [CD45(+), EPCAM(−), CD31(−), FAP(−)], epithelial [CD45(−) EPCAM(+), CD31(−), FAP(−)], leukocyte [CD45(−), EPCAM(−), CD31(+), FAP(−)] and fibroblast [CD45(−), EPCAM(−), CD31(−), FAP(+)] populations. GSE1133 consists of 79 human and 61 mouse tissue baseline gene expression microarray profiles. From this cohort, we selected the 79 human tissue transcriptional profiles. In addition to the NCBI cohorts, we have utilized both the GTEx and BioGPS portals. The RNA-seq data used for the analyses described in this manuscript were obtained from the Genotype-Tissue Expression (GTEx) portal (http://gtexportal.org/) version V6 in January 2016. This cohort consists of expression profiles from 53 human tissues across 8555 samples. The BioGPS database (http://biogps.org/) contains gene expression profiles across 745 samples representing a diverse range of primary human cells called the Expression Atlas of Human Primary Cells. This cohort was developed from combining a large number of publically available microarray datasets (745 samples, from over 100 separate studies) derived from human primary cells. Expression bar charts were plotted as median probeset values using GraphPad Prism version 5 for Windows.

### Risk assignment

The study design and filters applied at each step are outlined in Figure 1. The entire GSE39582 cohort was filtered by excluding stage I and stage IV patient transcriptional profiles, followed by any transcriptional profiles with missing relapse data resulting in a cohort of 460 transcriptional profiles. For the discovery subset, we removed patient transcriptional profiles which were censored to follow up prior to 36 months (unknown relapse data). Patients that relapsed prior to 36 months were classified as high-risk and patients with no relapse were classified as low risk, resulting in 372 transcriptional profiles. This discovery subset was further filtered to contain only transcriptional profiles from untreated patients, resulting in 177 transcriptional profiles. Three year relapse free survival analysis was performed on these 177 transcriptional profiles to determine relapse rate information across all CMS assigned tumors using GraphPad Prism version 5 for Windows. This 177 transcriptional profile subgroup was then further filtered to contain only CMS1 assigned transcriptional profiles resulting in 46 tumor transcriptional profiles, 6 assigned as high risk and 40 with low risk assignment (Figure 1).

### Transcriptional analysis

Partek Genomics Suite was used for dataset analysis. Differentially expressed probesets which had a fold-change +/− 1.5 fold and p-value<0.005 were defined using analysis of variance (ANOVA) of supervised risk groupings. For the purpose of clustering, the data matrices were standardized to the median value of probeset expression. Standardization of the data allows for comparison of expression levels for different probesets, which may not necessarily be on the same scale or at the same intensity levels. Following standardization, 2-dimensional hierarchical clustering was performed (samples x probe sets/genes). Euclidean distance was used to calculate the distance matrix, which is a multidimensional matrix representing the distance from each data point (probe set-sample pair) to all the other data points. Ward's linkage method was subsequently applied to join the samples and genes together, with the minimum variance, to find compact clusters based on the calculated distance matrix.

### Chemokine-like factor (CKLF) stratification

Tertile stratification was performed on the mean CKLF expression value from the three probesets used within the CMS1, MSI and complete cohorts. These values were classified as high, medium and low based on 1:1:1 sample distributions. In the case where there were uneven numbers for equal distribution, preference was given to equally distribute the high and low groups.

### Survival analysis

Survival validation was performed on the CMS1 (n=79) and MSI (n=60) transcriptional profiles from the 460 stage II/III transcriptional profiles with complete clinical information. Survival curves, comparing expression and treatment subgroups were plotted from patient details right censored at 36 months, to give 3-year relapse rates using GraphPad Prism version 5 for Windows. Cox Proportional Hazards analysis, using Stata version 11.2, was applied to evaluate recurrence-free survival according to CKLF gene expression levels within the CMS1 subgroup, within MSI tumors and in the entire cohort, prior to and after adjustment for age, sex, tumor stage and location, and receipt of adjuvant treatment. Stratified analysis was also conducted in untreated patients only, using the relapse data for the duration of follow up within the dataset (201 months maximum follow up). Categorical and continuous variables were compared between individuals with CMS1 tumors and the rest of the overall cohort using chi-squared tests and t-tests, respectively.

## SUPPLEMENTARY TABLES AND FIGURES


