# Supplementary material for: Stratified analysis reveals chemokine-like factor (CKLF) as a potential prognostic marker in the MSI-immune consensus molecular subtype CMS1 of colorectal cancer
Source: Oncotarget. 2016 May 2;7(24):36632–44. doi: 10.18632/oncotarget.9126 (PMC5095027; doi:10.18632/oncotarget.9126)
Supplement: Supplementary file 1 [file oncotarget-07-36632-s001.pdf]

SUPPLEMENTARY TABLES AND FIGURES

Supplementary Table S1: Cross tabulation of high and low risk tumors within each consensus subtype

Risk\Consensus group	CMS1	CMS2	CMS3	CMS4	Total
**High**	6	20	4	11	41
**Low**	40	58	14	24	136
**Total**	46	78	18	35	177
**% Relapse at 3-years**	**13%**	**26%**	**22%**	**31%**	

Supplementary Table S2: Cross tabulation of CMS1 patient profiles according to risk subgroup and BRAF mutational status

CMS1		
**Risk\BRAF Mutation**	**Mutant**	**Wild Type**
**High**	4 (15%)	6 (17%)
**Low**	22 (85%)	29 (83%)
**Total**	26	35

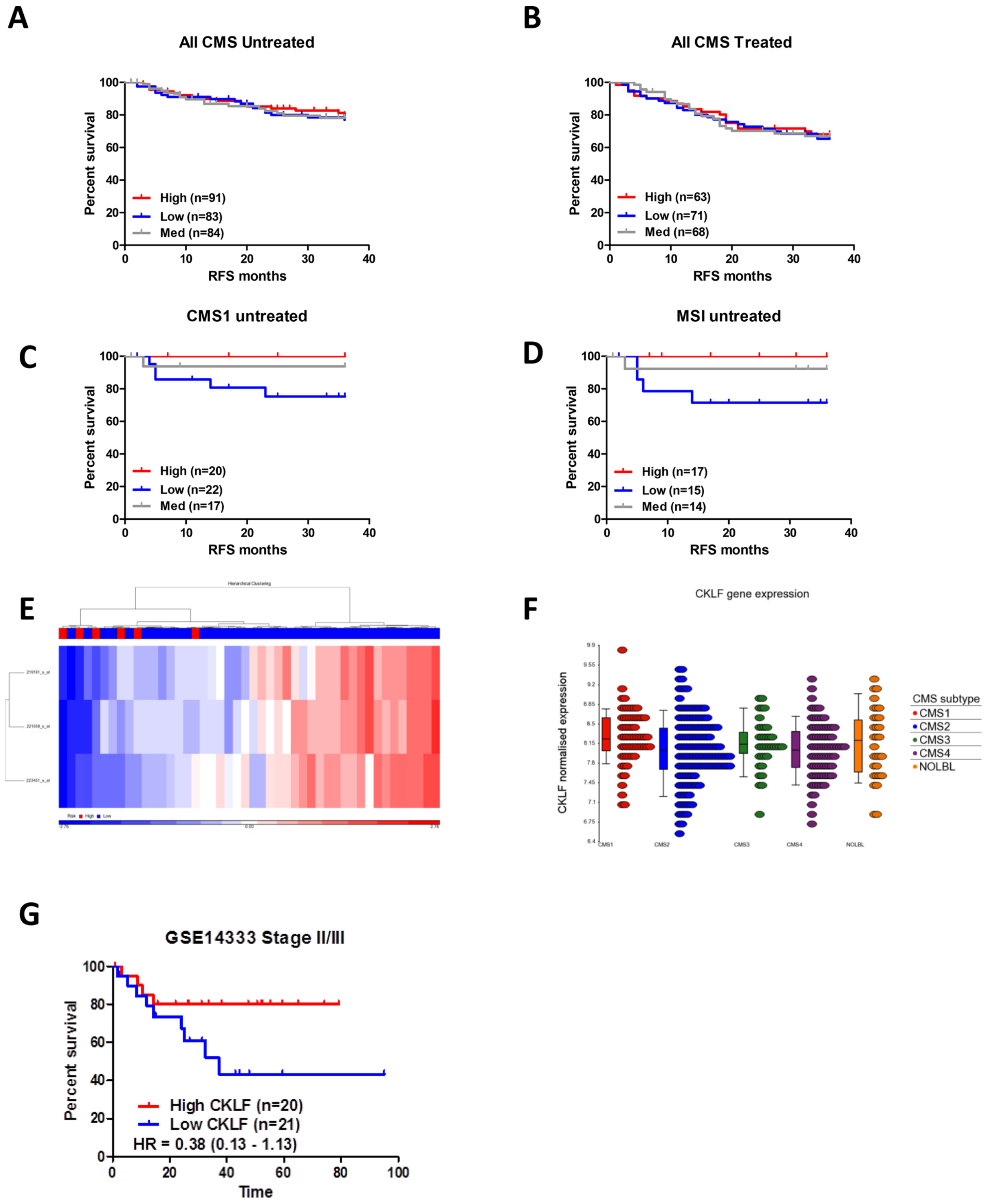

Supplementary Figure S1: Stratified risk analysis. A + B. Survival curve using Kaplan-Meier estimation comparing CKLF levels across all CMS in untreated (A) and treated (B) stage II/III CRC patients (GSE39582) **C + D.** Survival curve using Kaplan-Meier estimation comparing CKLF levels in untreated CMS1 (C) and untreated MSI (D) stage II/III CRC patients (GSE39582). **E.** Euclidean and Ward clustering of CKLF probeset expression values. Overlay bar indicated high risk (red) and low risk (blue) patient profiles. **F.** Dot plot with associated box plots representing CKLF gene expression levels across all consensus subgroups. **G.** Survival curve using Kaplan-Meier estimation comparing high v low CKLF levels across the 41 CMS1 stage II/III CRC patients (GSE14333).

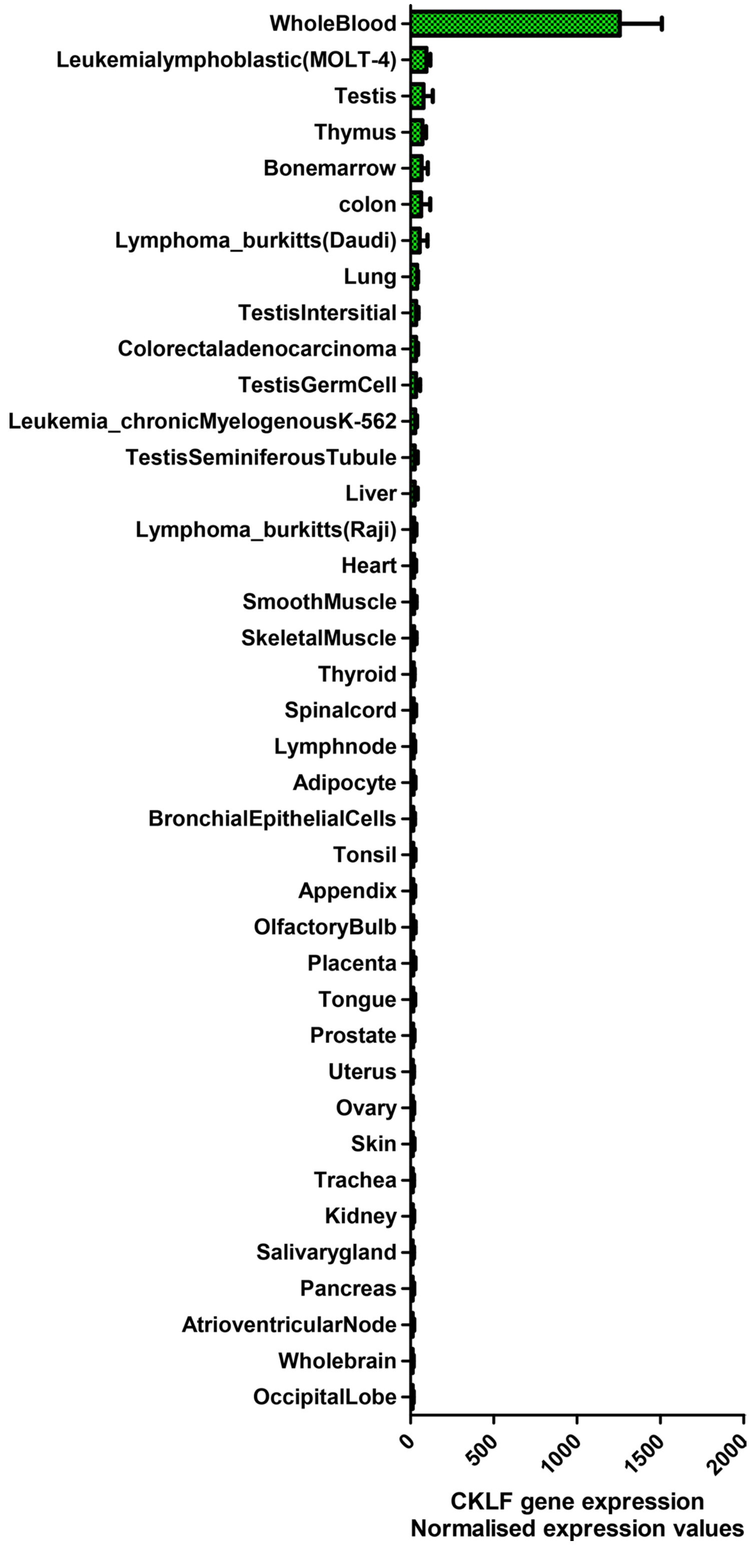

Supplementary Figure S2: CKLF probesets expression in human tissue profiles. Bar charts are shown as median of CKLF gene expression retrieved from GSE1133. This cohort consists of 79 human and 61 mouse tissue baseline gene expression microarray profiles, of which 39 tissues are represented. Error bars indicate standard deviation.

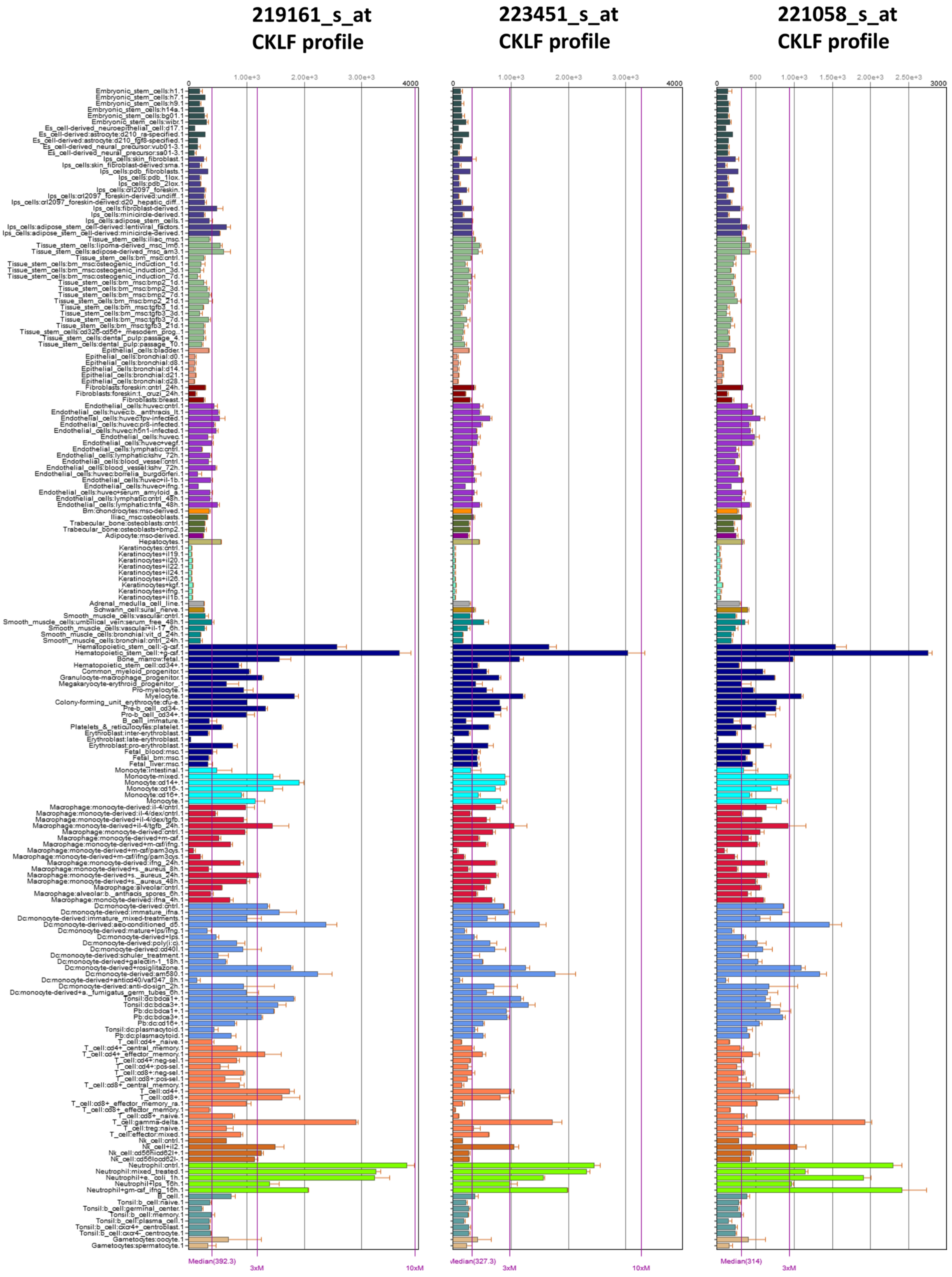

Supplementary Figure S3: CKLF probesets expression in human primary cells. Bar charts are shown as median of each individual CKLF probeset expression according to specific lineage and error bars represent standard deviation. Median gene expression levels for each probeset across all samples analysed are indicated. The data used for this analysis was obtained from the BioGPS gene annotation portal (http://biogps.org) in January 2016.
